# Hyaluronic Acid Scaffolds for Loco-Regional Therapy in Nervous System Related Disorders

**DOI:** 10.3390/ijms232012174

**Published:** 2022-10-12

**Authors:** Amel Djoudi, Rodolfo Molina-Peña, Natalia Ferreira, Ilaria Ottonelli, Giovanni Tosi, Emmanuel Garcion, Frank Boury

**Affiliations:** 1 Inserm UMR 1307, CNRS UMR 6075, Université de Nantes, CRCI2NA, Université d’Angers, 49000 Angers, France; 2 Nanotech Lab, Te.Far.T.I., Department Life Sciences, University of Modena and Reggio Emilia, 41125 Modena, Italy; 3 Clinical and Experimental Medicine PhD Program, University of Modena and Reggio Emilia, 41125 Modena, Italy

**Keywords:** hyaluronic acid, scaffolds, hydrogels, nervous system

## Abstract

Hyaluronic acid (HA) is a Glycosaminoglycan made of disaccharide units containing N-acetyl-D-glucosamine and glucuronic acid. Its molecular mass can reach 10 MDa and its physiological properties depend on its polymeric property, polyelectrolyte feature and viscous nature. HA is a ubiquitous compound found in almost all biological tissues and fluids. So far, HA grades are produced by biotechnology processes, while in the human organism it is a major component of the extracellular matrix (ECM) in brain tissue, synovial fluid, vitreous humor, cartilage and skin. Indeed, HA is capable of forming hydrogels, polymer crosslinked networks that are very hygroscopic. Based on these considerations, we propose an overview of HA-based scaffolds developed for brain cancer treatment, central and peripheral nervous systems, discuss their relevance and identify the most successful developed systems.

## 1. Introduction

Hyaluronic acid (HA), also known as hyaluronan, is a linear polysaccharide composed of disaccharide units containing N-acetyl-D-glucosamine and glucuronic acid [1,2,3]. Its molecular mass varies between 0.2 and 10 MDa and its physiological properties are governed by its polyelectrolyte and polymeric features, as well as by its viscous nature [1,2,3]. HA is a ubiquitous compound present in almost all biological tissues and fluids [1,2,3]. In the human organism, it is found in the extracellular matrix (ECM) of the skin, vitreous humor, cartilage, umbilical cord, CNS and the PNS [1,4].

HA has various applications. For instance, in cosmetology, it is used as an anti-ageing agent, in pharmaceutics and regenerative medicine as an excipient and constituent of scaffolds for drug delivery and regeneration and in biology as a component of in vitro models [5,6,7]. Research and medical grade HA samples are produced by biotechnological processes, mainly from bacteria or isolated from rooster combs [1,4]. HA is FDA-approved and generally recognized as safe (GRAS) for medical applications. Besides, HA can be administered topically, orally or by injection [1,2,3].

HA is a natural polymer present in the central nervous system (CNS) and peripheral nervous system (PNS) [7,8]. It is capable of forming a polymeric crosslinked network that exhibits a high water-absorbing ability, called a hydrogel [9]. HA polymers are biodegradable in vivo by free radicals and by vertebrates by specific enzymes from type I to type VI hyaluronidases [2,4]. HA biomimetic properties have motivated the development of HA-featuring devices for CNS and PNS disorders, displaying the following characteristics: injectability, biocompatibility, bioadhesion, controlled drug release and biodegradability [1,3,10].

HA-based systems are tunable. They are usually formulated with HA alone or in association with other biopolymers with biological properties such as chitosan, alginate and cellulose, or synthetic ones with thermosensitive and mechanical properties, for example poloxamers, polyethylene glycol (PEG) and polycaprolactone (PCL) [7,10,11,12,13,14]. Due to their versatility, research on HA-based polymers has increased over the past decade with potential applications for drug delivery and regenerative medicine [9]. The aim of this review is to highlight HA properties, summarize emerging in vitro and in vivo evidence of HA-based scaffolds developed for brain cancer treatment [15], the CNS [16] and PNS [17] and discuss the relevant results for the locoregional administration of these scaffolds (Tables S1–S3).

## 2. Pathologies of the Central and Peripheral Nervous System and Current Therapeutic Approaches

Pathologies of the nervous system are considered among the most difficult to treat, due to the complexity of the system and its numerous protective barriers that play a critical role in the brain’s metabolic activity as well as neuronal function [18,19]. At the same time, the occurrence of brain diseases like cancer, traumatic injuries and neurodegenerative diseases is currently increasing [20]. Notwithstanding the advances in research regarding novel therapeutic approaches to treat pathologies of the CNS, the majority of these diseases still lack an effective and permanent cure. Here, we introduce the main issues and current therapeutic approaches for three classes of brain pathologies: (i) cancer, particularly focusing on the most common and hard-to-treat brain cancer, i.e., glioblastoma [15]; (ii) traumatic brain injuries [16]; and iii) peripheral nerve injuries [17].

### 2.1. Glioblastoma and Gliomas

Grade IV glioblastoma (GB) and malignant gliomas are the most common form of brain tumors. They have an annual incidence of 5.26 per 100,000 people, accounting for 17,000 new cases each year [21,22]. Unfortunately, these kinds of tumors lead to a poor quality of life and prognosis for patients, with a survival median of 15 months after diagnosis [15,21]. In fact, GB is defined as a IV grade glioma, and it is the most lethal and frequent malignant primary brain tumor [21]. On contrary to other solid tumors, GB is highly invasive towards neighboring tissues in the brain, causing high rates of recurrence and tumoral transformation in healthy cells, although rarely metastasize to other organs [15,21,23].

Current FDA-approved therapy is based on a surgical resection of the majority of the tumor, followed by systemic chemotherapy and radiotherapy together with adjuvant therapy with temozolomide (TMZ), according to the Stupp protocol [24,25]. In spite of the undoubted advantages linked to this therapeutic approach, in 90% of the cases patients experience a recurrence after the first surgery [15,26]. This common issue is mainly due to a specific population of cancer stem cells, called tumor-initiating stem cells, which display a high tumorigenic potential and often remain intact even after surgery and chemotherapy [23,27]. Thus, their ability to proliferate in an asymmetric way leads to the relapse of GB, but few therapeutic options are available in this scenario [15,21,23,28].

To reduce the risk of a recurrence, several therapeutic approaches are now under investigation (Table S1). Of particular interest is the possibility to develop peptide- and cell-based vaccines that specifically target GB cells leading to their death by stimulation of the immune system [26]. More recently, CAR-T cells have also gained increasing interest from scientists thanks to their high potential for the treatment of GB [29]. Other approved molecules for the treatment of GB include carmustine, lomustine and even monoclonal antibodies such as bevacizumab [30]. Another innovative approach is represented by nanomedicines; in fact, the particular tumor microenvironment and surface characteristics of GB cells allow for the specific targeting of drug-loaded nanosystems that can release cytotoxic drugs in tumor cells with low off-target effects [31,32]. Despite the several advantages that these innovative therapies may involve, few of them have reached the market. Besides, one approach is represented by Gliadel^®^ [33,34,35]. Developed in early 2000, Gliadel^®^ consists of polymeric wafers loaded with carmustine. They are approved to be implanted into the resected cavity after surgery and are able to release the drug for up to 3 weeks, thus reducing the risk of GB setback and promoting a better prognosis for patients [35]. However, this treatment only produces modest effects as the released drug can only penetrate 1–2 mm into the surrounding brain tissues, preventing the complete killing of residual GB cells, which may reside more than one centimetre away from the resection margin. Moreover, the shape of the scaffold does not quite fit the resection cavity border and induces some side effects in many patients according to various clinical trials [36,37,38].

### 2.2. Traumatic Brain Injuries

Traumatic brain injuries (TBIs) are on the rise, not only in the elder population, where almost 500 out of 100,000 people over 80 years old are estimated to suffer from this pathology each year, but also in children, with an annual incidence of almost 500,000 cases among children aged 0–14 [16]. In particular, TBIs are one of the major causes of impairments in young adults, and for this reason, they represent a huge healthcare burden [39]. After the traumatic event, many phenomena can occur in the brain, both at physical and chemical levels [16,40]. In particular, the first phase after the trauma is generally considered crucial to determine the development of secondary damage, as it can include haemorrhages, disruption of the Blood Brain Barrier (BBB), high levels of inflammation, with consequences such as seizures, hypoxia, ischemic areas, and edema [41]. All these events, if not timely treated, may lead to metabolic failure, eventually leading to critical impairments or even the death of the patient [16,40].

Therapeutic approaches approved in the case of TBIs are generally linked to the specific case and history of the disease [16]. Considering physical and pharmacological approaches, these may include surgery to reduce edema-induced intracranial pressure, the administration of neuroprotective agents, antioxidants to reduce free radical production and anti-inflammatory drugs; also, other therapies can involve hypothermia, the regulation of blood flow dysregulation and ischemia, the regulation of ion homeostasis and cytoskeleton stabilization [42]. All of these pharmacological strategies, though, present a common limitation due to their fast clearance, thus resulting in a hampered prolonged release and the need for several therapeutic systems [41]. At the same time, surgery often includes craniotomy and cranioplasty, calling for the need to develop biocompatible materials that can substitute physiological tissues and promote recovery [43,44]. Both of these issues have been under investigation in recent years, leading to the design and formulation of biocompatible scaffolds that allow the prolonged release of therapeutics, along with the promotion of tissue curing [45,46]. In fact, many scaffolds have been developed, using different materials and production techniques (Table S2). In particular, hyaluronic acid-based scaffolds will be discussed in part 5.

### 2.3. Peripheral Nerve Injuries

Peripheral nerve accidents are not unusual situations, with a vast range of symptoms depending on the severity of the trauma and the nerves involved [17]. Although a lot of information exists on the mechanisms of damage and regeneration, reliable treatments that allow for complete recovery are rare [17,47,48].

Peripheral nerve accidents can imply various challenges to patients, starting from moderate pain to life-long impairment (Table S3). Seddon pioneered nerve accidents classification, by identifying three primary classes based on the degree of demyelination and the amount of damage to the axons and the connective tissues of the nerve [17]. The mildest shape of harm is referred to as neurapraxia, described by focal demyelination in the absence of harm to the axons or the connective tissues [17,49]. Neurapraxia generally happens as a consequence of moderate compression or the traction of the nerve and results in a lower conduction velocity [17,49,50]. Depending on the severity of the demyelination, the consequences can change from asynchronous conduction to conduction block, resulting in muscle weakness [17,51]. The subsequent stage is referred to as axonotmesis, which includes direct harm to the axons as well as focal demyelination, but the continuity of the nerve’s connective tissues is preserved [17,50]. The most extreme form of damage is referred to as neurotmesis, involving the complete transection of the axons and total nerve discontinuity [17,50,51].

## 3. Routes of Administration of Active Pharmaceutical Ingredients (API) into the CNS and Challenges

### 3.1. BBB and Limits of Drug Diffusion

The blood vessels that supply the central nervous system (CNS) have unique properties, known as the blood-brain barrier, that allow them to tightly regulate the movement of ions, molecules, and cells between the blood and the brain. This precise control of CNS homeostasis enables proper neuronal function and also protects neural tissue from toxins, pathogens and changes in barrier properties. This is an important part of the pathology and progression of various neurological diseases. The physiological barrier is coordinated by a series of physical, transport and metabolic properties possessed by the endothelial cells (ECs) that make up the vascular wall, and these properties are regulated by interactions with various vascular, immune and nerve cells [52,53,54].

CNS vessels are continuous, non-windowed vessels, but they also contain many additional properties that allow a tight control of the movement of molecules, ions and cells between the blood and CNS [53,55]. This highly restrictive barrier capacity allows the endothelial cells of the BBB to tightly regulate CNS homeostasis, which is essential for proper neuronal function, as well as for the CNSs protection against toxins, pathogens, inflammation, injury and disease. The limited nature of BBB is a barrier to drug delivery to the CNS and therefore great efforts have been made to create methods to modulate or disrupt the BBB for therapeutic drug delivery. The main route used to administer drugs is the intravenous route, and the BBB is a limiting factor. Hence, to overcome this hurdle, locoregional direct routes have been used [56,57].

**Figure 1:**
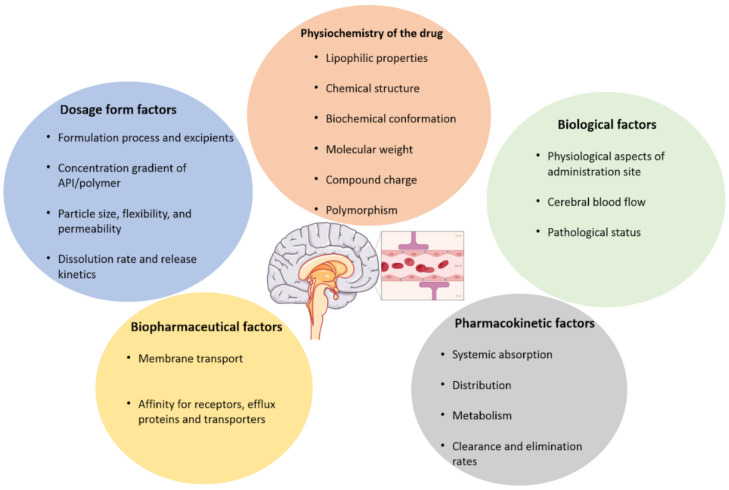
Parameters affecting the ability of drugs to cross the blood brain barrier (BBB). This figure illustrates the factors that condition the BBB crossing. Among them, we can find dosage forms factors like the particle size, flexibility, the release profile, the excipients contained in the formulation. Other parameters, such as physico-chemistry of the drug, like the molecular mass, the chemical structure or the charge of the drug. Adding to these parameters, the biological and biopharmaceutical factors such as the cerebral blood flow, the membrane transport type, and the physiological characteristics of the target site. Finally, pharmacokinetics can also influence the drug from passing the BBB.

**Figure 2:**
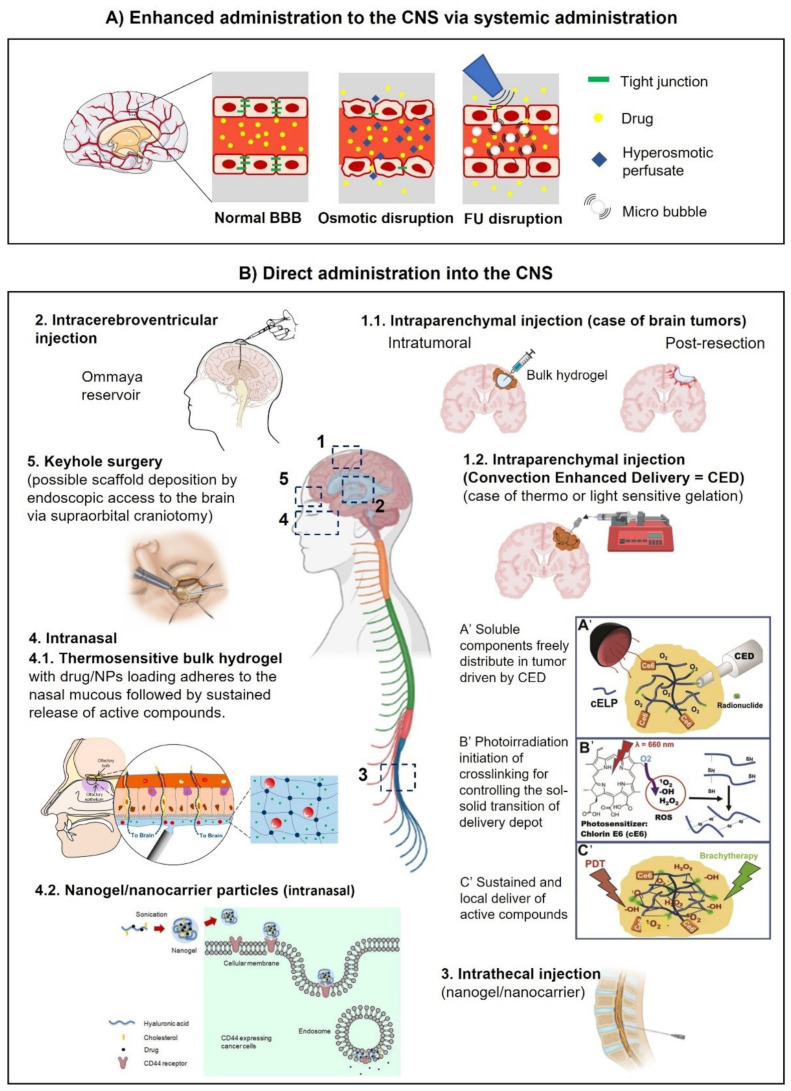
Routes of administration into the CNS. (**A**) Enhanced administration of molecules and nanobodies by osmotic or focused ultrasound (FU) disruption of the BBB. (**B**) Direct administration: 1.1. Intraparenchymal injection. A brain tumor case is schematized where hydrogel injection might be performed intratumorally or after resection around the cavity edges. 1.2. Intraparenchymal injection assisted by CED (convection-enhanced delivery). Depicted is the case where soluble compounds are evenly distributed by CED within the tumor before a gelation reaction is induced by photoirradiation. The resulting embedded gel can be used as a platform for the sustained release of active compounds [86]. 2. Intracerebroventricular administration of drugs directly into the cerebrospinal fluid (CSF). The Ommaya reservoir consists of a catheter connected to one lateral ventricle and a reservoir implanted under the scalp [91]. 3. Intrathecal injection. Lumbar puncture showing the direct administration of a drug directly into the CSF. 4. Intranasal delivery. 4.1 Intranasal application of modified HA in the nasal endothelium. Upon in situ polymerization the generated patch might be used as a reservoir for sustained release of compounds [62]. 4.2 After permeation of the nasal barrier, HA nanogels may be used to enhance intracellular trafficking of drugs in CD44 expressing cells [87]. 5. Keyhole surgery [43,90] might be used as an alternative access route to the implantation of hydrogels into the brain. Credits: Figure 2B-1.2: reprinted from Mukerji et al., 2022 [86], with permission from Elsevier. Figure 2B-2: reprinted from the public domain access at https://en.wikipedia.org/wiki/Ommaya_reservoir#/media/File:Ommaya_01.png (accessed on 5 October 2022) Lynch PJ. Figure 2B-4.1: reprinted from Kiparissides et al., 2022 [62], with permission from ACS. Figure 2B-4.2: reprinted from Wei et al., 2022 [87], with permission from ACS. Figure 2B-5: reprinted from Core Techniques in Operative Neurosurgery 2022 [91], with permission from Elsevier. Central and additional figures were created with BioRender.com.

Figure 1 shows the main drawbacks of the BBB. Among them, biological factors such as cerebral blood flow, the physicochemical properties of the drug, like the chemical and biochemical structure, the compound charge and molecular mass, the dosage form parameters, like the formulation process used, the particle size and release kinetics. Other factors affecting drug transportation through the BBB are pharmacokinetics (ADME and clearance types), and biopharmaceutical factors like the membrane transport and affinity of the drug for cell receptors. Several administration types have been developed in the following parts, among them are the enhanced systemic administration, intranasal administration, convection- enhanced delivery and the intracerebral route (Figure 2).

### 3.2. Enhanced Systemic Administration

One of the main challenges in the treatment of CNS-related diseases is the bioavailability of the API in the damaged tissue. Systemic administration faces the constraint of the BBB, overall biodistribution and clearance from the body [52,56]. To overcome such limits, techniques such as aortic injection and the enhanced permeation of the BBB by differential osmotic pressure [19] and cavitation generated by a high intensity ultrasound combined with the intravenous administration of microbubbles [58,59,60], have been investigated (Figure 2A). Although these techniques have the potential to increase the diffusion of active compounds, the accumulation into the desired site could be compromised, while at the same time the clearance from the CNS tissues and circulating blood is held. Thus, the therapeutic agent needs to be continuously administered on a planned basis in order to avoid considerable systemic toxicities [19].

### 3.3. Intranasal Administration

Intranasal administration consists in the penetration of APIs into the CNS through the nasal barrier. The intranasal pathway can deliver therapeutics directly from the nasal cavity to the brain via the olfactory and trigeminal neurons. The intranasal route is made up of two routes, one intracellular and one extracellular [61]. The intracellular process begins with olfactory sensory cell endocytosis, which is followed by axonal transport to the synaptic clefts in the olfactory bulb, where the drug is exocytosed [61]. This transsynaptic process is replicated by olfactory neurons, allowing the medication to be distributed to different brain areas [61]. Drugs are carried directly into the cerebral spinal fluid via the extracellular method by first going via the paracellular space over the nasal epithelium, then through the perineural space to the brain’s subarachnoid space [61].

**Figure 3:**
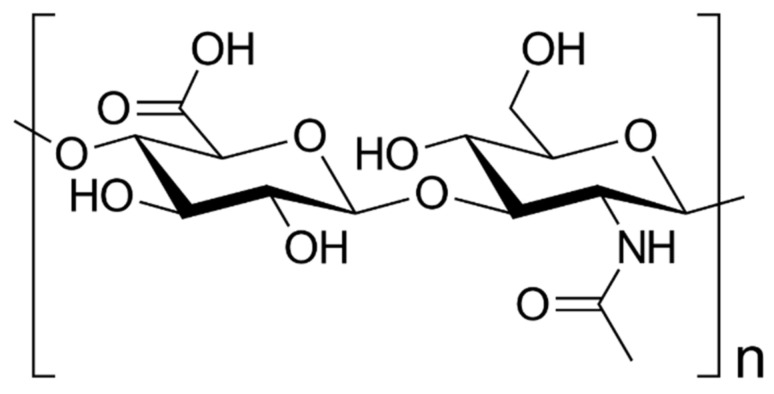
Hyaluronic acid chemical structure. HA structure consists on an anionic glycosaminoglycan non-sulfated repeating disaccharides of β4-glucuronic acid (GlcUA)-β3-N-acetylglucosamine (GlcNAc).

**Figure 4:**
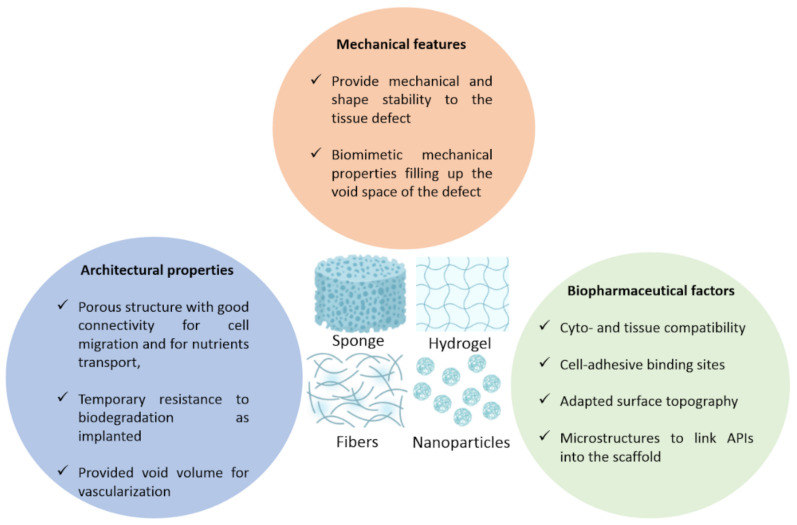
Scaffold specifications depending on various parameters. The specifications that are usually expected in scaffolds whether it is sponges, hydrogels, fibers or nanoparticles, that closely mimic the brain’s ones like shape stability and close biomechanical features. Moreover, architectural properties that are usually linked to porosity, connectivity and a transient stability upon degradation. Finally, biopharmaceutical factors like cyto and tissue compatibility, binding sites, microstructure of the scaffold.

One of the limitations of this route is the availability of the compound in contact with the nasal mucous membrane. Hydrogels may function as a reservoir for a prolonged release of APIs in the nasal route towards the CNS [62] (Figure 2B-4). In other works, the development of nanobodies have been recently explored for the intranasal delivery of vaccines encapsulated in a nanogel [63], temozolomide administration for glioblastoma treatment [64], poorly soluble drugs such as simvastatin [65], and intranasal clozapine-loaded Technetium-99m-labeled mixed micelles for the treatment of schizophrenia [66]. Overall, although under investigation, this route may offer a non-invasive approach to the delivery of APIs in combination with an appropriate encapsulation in nanoparticles, including nanogels.

### 3.4. Direct Administration into the CNS Compartment: The Intracerebro-Ventricular and Parenchymal Routes

Since the majority of GB recurrences arise within the margins of the resection cavity, intraoperative loco-regional therapies (e.g., ascribed to differentiation, chemoattraction-trapping, immunostimulation strategies) become more and more relevant [67]. Invasive techniques that include the physical disruption of the BBB have also been investigated with the aim of reaching the damaged area more directly while reducing the total dose needed. For example, intracerebroventricular and intrathecal injections have the advantage that the drug is administered in the fluid compartment that is already in the CNS, hence the drug can more quickly reach the area of interest [18,68] (Figure 2B-2,3). Moreover, administration via this route can be continuous by depositing a catheter connected to a pumping system [69]. Both routes of administration have the advantage that the main BBB step is bypassed, contrary to a systemic administration [68,70]. However, the diffusion into a distal site within the CNS can still be reduced due to other biological barriers, and the active compound may affect healthy tissues causing neurotoxicity or off-target effects [70,71,72].

A more direct approach consists of the injection of the drug directly into the damaged site. This approach, referred to as an intracerebral parenchymal injection (Figure 2B-1), is relevant in the case of macroscopic lesions such as visible brain tumors and brain ischemia. The challenge of this strategy is the internal fluid pressure that can cause a reflux of the administered substance if performed in a single shot. Therefore, an additional force is needed to enhance the distribution of the administered substance. This is the principle of the strategy called convection-enhanced delivery (CED) in which a differential pressure is applied by means of a pumping system connected to a catheter that delivers the load gradually as the molecule of interest is locally and regionally spread into the interstitial space by convection and diffusion [73,74] (Figure 2B-1.2). CED has various benefits, such as a bulk flow-controlled process, bypassing the BBB, targeted delivery, and achieving reproducible diffusion [75].

These techniques have been used for the administration of soluble drugs or colloidal dispersions of nanoparticles, sometimes delivering radiopharmaceuticals with promising results [63,64]. Nevertheless, although they offer a more loco-regional delivery, the absence of a reservoir that gradually releases the active compound represents a limitation. CED may function as a reservoir strategy for gradual delivery, however the optimal regime depending on drug formulations needs to be established in order to diminish potential risks in the neurological status of the patient linked to an accumulation of the drug and/or the increase of the intracerebral pressure [76].

### 3.5. Delivery of Scaffolds as Prolonged Releasing Platforms

A novel approach consists of the use of implantable devices which can ensure a sustained release of the active compound without the need for repeated injections. These releasing platforms are often formulated as hydrogels, fibers and porous scaffolds [77,78,79]. The success of these systems has already been demonstrated, as in the case of Gliadel^®^ wafers: when implanted in the margins of the glioblastoma resection cavity, they allow for a sustained release of carmustine [33,34,35,38]. However, the main disadvantage is that the wafers do not fit perfectly into the space and contact with the brain margins could be compromised [36].

Hydrogels, by contrast, offer the possibility of an adequate fitting into cavities and are very attractive as delivery systems. Indeed, they are of special interest in zones where the tissue has been resected, such as tumors, brain and spinal cord injuries that undergo surgery (Figure 2B-1.1). The versatility of such systems relies on the feasibility of injection as consolidated hydrogels or as soluble components that are sensitive to temperature or radiation to become stable hydrogels in situ [77]. The selection of either strategy is dependent on the application. For example, to fill a superficial cavity, thermosensitive soluble components would be easier to deposit with a subsequent instantaneous reaction upon a change in temperature, whereas the endoscopic delivery of thermosensitive compounds may not be suited for long distances that could result in the gelation of the solution in the tubing system [80,81,82]. On the contrary, the injection of a consolidated hydrogel may be limited to the rheological behavior and nature of the components. For instance, changes in structure or properties after injection and degradation due to hydrolysis are variables that could constrain their application [81,83,84,85].

### 3.6. Delivery of Hydrogels by CED

The main advantage of the use of convection for delivery is the control of the time-space distribution of the load. While CED has been investigated since the early ‘90s on the liquid formulations for intracerebral injections, little research has been made with regard to the delivery of hydrogels [77]. For instance, Mukerji R et al., 2015 [86] developed a system of soluble elastin-like polypeptide (ELP) containing periodic cysteine residues which were conjugated with chlorin-e6 (Ce6) as a photosensitizer. The soluble peptide was distributed in vivo into a solid tumor by CED to allow an even distribution within the whole tumoral mass (Figure 2B-1.2). Upon photon stimulation, the produced ROS allowed disulfide crosslinking across the cysteine chains that eventually originated a reticulated network forming a hydrogel embedded in the tumor [86]. This strategy allowed to overcome the rheological constraints that can impose the injection of viscous materials. Additionally to photoirradiation, a thermal, enzymatic [62] and sonication [87] activation of the gelling of the injectable liquid mix might be explored in combination with CED.

### 3.7. Potential Hazards and Challenges

**Figure 5:**
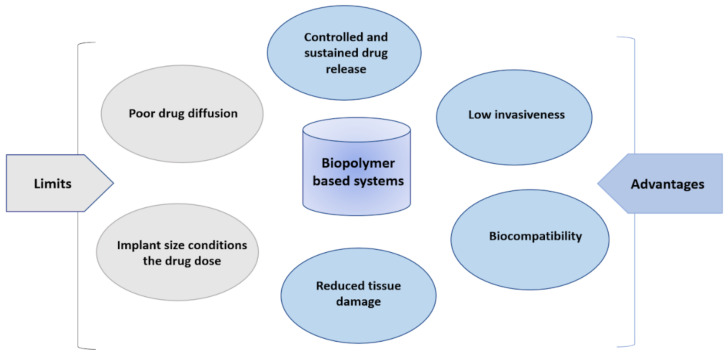
Characteristics of polymer-based devices for central nervous system delivery.

The fact that the direct administration of drugs into the CNS is an invasive approach, can cause patient side effects such as edema, infection and neuron damage [68,88,89]. The safety hazards, drawbacks and relatively high prices held up their applications as standard therapeutic strategies for those CNS diseases with relatively long disease processes and needing repeated administration [89]. Reducing the invasiveness of the procedure by exploring minimally invasive surgery such as keyhole surgery [43,90], for the deposition of catheters or the injection of scaffolds (Figure 2B-5), accompanied with image-guided surgery, might result in a benefit to the patient. 

Intracerebral drug delivery is a method of passing through the BBB and other mechanisms that limit drug distribution in the brain, allowing high concentrations of a drug to enter the central compartment. Factors that affect the efficacy and safety of this route of administration are osmotic pressure, pH, volume and the presence of preservatives and drug vehicles being administered [89]. Physicians should be aware of the ongoing pathology process and the patient’s neurological status, as well as the physicochemical properties of the associated drug when prescribing for intracerebral administration. High suspicion parameters should be maintained when monitoring patients for adverse drug events after administration [89].

## 4. Therapeutic Relevance of HA-Based Scaffolds in the CNS

### 4.1. Composition, Biological Properties and Mechanical Properties of CNS ECM

The extracellular matrix (ECM) plays a diverse role in several physiological and pathological conditions [92]. In the brain, the ECM is unique in both composition and function. In addition, almost every cell in the central nervous system contributes to various aspects of this complex structure [93]. ECMs in the brain, rich in proteoglycans and other small proteins, aggregate into distinct structures around neurons and oligodendrocytes [94]. These special structures play important roles in normal brain functions such as learning, memory and synaptic regulation [92,93].

Chemical modification further diversifies the processing and manufacturing techniques that can be used to create 3D HA scaffolds [95,96,97]. By easily changing the treatment method, HA hydrogels, granular hydrogels (microgels), electrospinning fibers, and HA-based composites can be formed [77,98,99,100]. Various types of HA scaffolds have their own characteristics which offer several benefits to CNS regenerative medicine [79].

Murine and human brain ECM stiffness and the physico-chemical specification of these composite scaffolds need to be associated. Indeed, the brain ECM is mainly composed of HA and gives this tissue a softness property [93]. Moreover, the Young modulus, in the region of the hippocampus, cerebellum and the cerebral cortex, varies from 0.5 to 10 kPa according to the region studied in murine brain [101]. It can be around 2 kPa for a healthy brain and go up to 20 kPa for a tumor affected brain, but in more rigid regions such as the dura mater, it can reach a very high Young moduli of 32 MPa and 62 Mpa [101,102,103,104]. Thus, formulating scaffolds with the Young modulus in this range is possible, especially for systems like hydrogels, sponges and fibers [77,78,105,106,107,108]. It also has been shown that in a healthy brain vs. an injured brain, the stiffness of the tissue can either increase or decrease according to the brain region and the type of injury (neurodegenerative disease or cancer) [109,110,111,112,113]. Various scientific projects on brain tissue mechanics have concluded that the brain is a very soft tissue, non-linearly viscoelastic solid material with a very low linear viscoelastic strain interval, around 0.1 to 0.3% [105].

Brain tissue is made up of white and grey matter, and different areas of the brain are composed of various proportions [62,114]. White matter mainly consists of myelinated axons from nerve fibers; the grey matter is driven by unmyelinated axons and perikaryons [105,106]. It is necessary to understand the mechanical properties of brain tissue, as the brain is so well isolated from mechanical damage under normal circumstances [109]. Mechanical factors are thought to play a role in many diseases, including brain development, but brain mechanics has been most often studied to understand stressed conditions, in an indirect or direct way [105,111,112].

### 4.2. Physico-Chemistry of HA-Based Semi-Solid Dosage Forms

The brain has an Hyaluronic acid (HA)-enriched ECM, in a healthy one, and a high molecular weight (>10^6^ Da). As shown in Figure 3, HA is a negatively charged and non-branched GAG (Figure 3). 

HA chains function as the tissue center of the ECM (Figure 3) and interact with proteins and PGs via a small linker protein called HABP to form a hydrogel-like network [92,93]. HA is upregulated in GB tumors and contributes to many phenotypic changes associated with cancer progression, including early tumor development, cancer cell proliferation, infiltration, drug resistance and post-treatment recurrence [104,115]. In addition, hyaluronidases, HA synthases, HA receptors and some HABPs are overexpressed. Co-overexpression of these factors may be implicated in GB invasion and treatment resistance [114,116,117].

For better mechanics, biomimetism, biocompatibility, sustained release and smart properties such as thermosensitivity, poloxamer and HA are well-studied and great candidates for semi-solid dosage forms and, more precisely composite hydrogel formulations [77,84].

Hydrogels are 3D water-swelling polymer networks formed by chemical and/or physical interactions. The main advantage of using hydrogels in tissue engineering constructs is that they are not only easy to process and mold, but also have the ability to adjust mechanical and biochemical properties to mimic soft tissues [77,118]. HA is interesting in CNS, brain cancer and peripheral nerve engineering because of its natural origin, non-immunogenicity, high biodegradability by hyaluronidase and hydrolysis, porosity, biocompatibility, neuronal differentiation and neurite outgrowth capacity [8,79] (Figure 4). Hydrogels have received a great deal of attention due to their unique properties such as an excellent biocompatibility, high water content and the ability to decompose into safe products, and are widely used in various biomedical applications such as regenerative medicine, aesthetic medicine and drug delivery [9,119].

Poloxamer is a family of synthetic nonionic triblock copolymers in which the central hydrophobic block of polypropylene oxide is sandwiched between two hydrophilic blocks of polyethylene oxide. Polyethylene oxide copolymers [67,94,95], which amongst them are poloxamer 407 (P407) hydrogels, exhibit interesting thermal properties and are attractive candidates for formulations, especially in combination with HA. P407 is a temperature-responsive polymer that is cold and liquid [85]. Aqueous polymer solutions gel as the temperature rises. P407 is considered to be one of the safest polymeric materials for the production of thermal hydrogels. It has an excellent biocompatibility and injectability and is used in various fields of tissue engineering [120,121,122]. Hydrophobic domains in the chemical structure of P407 are useful for retaining poorly water-soluble compounds. Various active substance-containing hydrogels based on P407 were developed and characterized as a function of active substance concentration. Since then, hydrogels have been used as a controlled drug delivery system to promote the local, sustained and long-term release of APIs, thereby reducing dosing frequency, avoiding side effects and complying with low doses. The most widely studied environment-sensitive systems are temperature-sensitive hydrogels, where physical entanglement, hydrogen bonds and hydrophobic interactions are key functions that make up the crosslinks. There are two distinct types of thermal hydrogels that gel by cooling below the upper critical gelation temperature (UCGT), such as agarose, or by heating above the lower critical gelation temperature (LCGT), such as poloxamer [123,124].

Hydrogels with LCGT behavior and sol-gel transition at 37 °C can be loaded under mild conditions (temperature ≤ 37 °C), making them very popular in the biomedical field as carriers for cells, drugs and biomolecules [119,125]. The solubility of hydrophobic parts decreases when aggregated, to reduce the interaction of PPO blocks with the solvent used. Poloxamer is well-known for its thermal responsiveness, biocompatibility and low toxicity; P407 is widely used in smart drug delivery and in various formulations such as ophthalmic, nasal and other parenteral galenic forms [120,121,122]. When P407 gel is used alone, it rapidly loses its gelling ability after being diluted in a water-enriched environment. Blending P407 with other polymers such as hyaluronic acid or molecules is a solution to improve drug loading; these composite hydrogels are widely developed in the literature [95,126,127,128]. For example, P407-based hydrogels are widely used to encapsulate some small molecule drugs, such as ketorolac, metoprolol and doxycycline, which have a molecular weight (MW) of less than 500 Da. They are also suitable for achieving the optimal release of proteins to facilitate the transfer of water molecules and release proteins or other compounds with a Mw > 500 Da such as Urokinase and Rutin [126,129].

## 5. HA-Based Specific Device for Various Applications

Hydrogels are hydrophilic polymer networks that swell in water or body fluids. Recently, in situ gelation systems based on various synthetic and natural polymers have been extensively investigated for biomedical applications due to their ability to efficiently encapsulate cells and bioactive molecules, to be a minimally invasive injection and be easy to form in any desired defect shape, in addition to some advantages of typical hydrogels, including a high water content similar to the extracellular matrix (ECM) [77,92,93], controllable physico-chemical properties (rheology and injectability) and biocompatibility [119,125]. When hydrogels are developed by covalent crosslinking, they form chemical or permanent gels. On the other hand, when physical bonding between molecules produces hydrogels, they form physical gels and they are usually reversible [81,85,130]. Hydrogels are polymeric mesh networks that have the ability to bind a high amount of water [77,118,131]. It is a semi-solid dosage form that is usually used for transdermal, subcutaneous, intra-articular, ophthalmic, nasal, vaginal and rectal administration routes [118,131]. The intracerebral route is the route that will be discussed in the following part; it is of high interest for brain cancer and central nervous system therapeutic strategies [68,88,89].

Unlike scaffolds that have a particular shape before being applied, injectable scaffolds are injected into the defect area and then acquire their shape in situ [107,132,133] (graphical abstract). This function allows solidifying precursor scaffolds and cell mixtures to be site-specifically delivered into cavities and defects with an irregular aspect, in a less invasive manner than transplantation [134,135,136,137].

Several studies deal with HA-based devices composed of other biopolymers such as heparin, silk fibroin [107], chitosan [12,132,138,139,140,141], collagen [132,142,143] and alginate [11,144], or synthetic ones like poly(methylvinylether-alt-maleic acid [133], polycaprolactone [145,146], PDLLA [147], PEG [148] and poloxamer [95,126,127,128]. These composite scaffolds made of natural polymers are biocompatible systems that exhibit interesting properties for tissue engineering applications such as structure, porosity, stiffness and correct controlled degradation rates [14,149]. Natural macromolecules exhibit abilities for site-specific cell adhesion, and given that HA is the main component of the brain ECM, neural cells can interact with a matrix which mimics their natural environment [3,10,14]. In the most recently presented studies, these systems have been developed for various applications such as GB treatment, peripheral nerve regeneration and brain tissue engineering (Tables S1–S3).

Since they exhibit shear-thinning properties in rheological studies as well as when being injected, some hydrogels have been applied as fillers in nerve guide channels (NGCs) in order to induce the regeneration of peripheral nerve tissue [48,141,150,151]

Other lyophilized or dried biopolymer scaffolds have been administered locally for drug delivery, cell encapsulation or cell tissue colonization [107,133,137,140,146,152,153]. They have various applications such as cartilage, brain tissue, bone regeneration or treatment. They provide structural support for cell attachment and subsequent tissue growth. They consist of biological substitutes to restore, replace or regenerate defective tissues, and mimic a tissue-specific ECM [113,114,115,116].

Biomaterial scaffolds are one of the most important factors in promoting cell differentiation and proliferation to form new tissues of interest. Tissue-engineered scaffolds must have multiple functions, including proper porosity, optimized mechanical properties, well-controlled biodegradability, non-destructive sterilization and biocompatibility with treated tissue [154,155,156].

Electrospun fibers have applications such as drug delivery, tissue engineering, wound dressing and cosmetics [98,108,157]. Electrospinning is a type of electrospray process and consists of strong electrical forces that overcome the weak surface tension of polymer solutions at specific thresholds to emit a jet of liquid that can be rooted in the process of electrospray, forming small solid polymer droplets and/or fibers [78,98,108,157,158].

A nanogel is a three-dimensional nano-sized hydrogel material composed of a cross-linked swellable hydrophilic polymer network with a high water storage capacity, without actually dissolving in an aqueous medium. Nanogels can be made from a variety of natural, synthetic, or polymer combinations [159,160,161,162].

Nerve guidance conduits are tubular devices made of wide-range biomaterials that guide axial regeneration from the injured proximal nerve to the distal stump. It is a type of bridging that helps avoid nerve grafting and nerve healing, which are both limited [47,48,163].

### 5.1. Scaffolds Specifications

The scaffolds described in the Tables S1–S3 must show some features, firstly architectural features, in other words, a suitable porosity in case of cell encapsulation or a system requiring cell trapping and migration, short-term resistance to biodegradation and a void volume for blood vessels ramification (Figure 4). Secondly, mechanical properties, like a shape stability and biomechanical mimicking of the treated tissue, for instance by having a close Young modulus, also called the elastic modulus (kPa). Lastly, the biopharmaceutical features required are cyto and histocompatibility, surface topography, cell-anchoring sites and microstructures for the drug to bind into the scaffold, which is helpful for controlled drug release.

### 5.2. HA-Based Scaffolds for Glioma Application

Various studies have managed to develop systems for GB treatment that exhibit properties close properties as the ones shown in Figure 4. The first authors to provide anti-inflammatory and anti-cancer natural molecules using HA nanohydrogels were [164]; it is a combination of quercetin and temozolomide for the treatment of GB. In this study, it was shown that quercetin nanohydrogel promotes the preferential uptake of CD44 and significantly enhances the therapeutic effect of temozolomide in GB cells, possibly through an anti-inflammatory mechanism [164]. Moreover, other results suggest that doxorubicin-loaded modified hyaluronic acid nanogels are an excellent candidate to effectively achieve glioma targeting [162].

Another study showed that the efficacy of HA-coupled micelles was increased by a stronger inhibition of glioma proliferation and the induction of apoptosis. Overall, these findings demonstrated the benefit of GB-associated chemotherapy using HA-coupled micelles [96]. Additionally, HA-CF/CB hydrogel has the potential to be a strong candidate for drug delivery vehicles, especially for the treatment of GB. Injections of DOX -loaded HA-CF/CB hydrogel into GB ex vivo human tissue samples showed an efficient attachment of the gel within diffusion and the release of the compound into the surrounding tissue [165]. Another system for GB treatment consisted of a polymer-drug conjugate releasing DOX which has shown a decrease in cell viability and the inhibition of the tumor growth [166]. Drug delivery systems composed of HA, intended for GB treatment, have also shown to be promising in vitro on GB cell lines and in vivo on mice and rat models results, in terms of bioperformance, biocompatibility, biomimetism and the controlled drug release of DOX, PXL and/or TMZ [113,162,167,168,169] (Figure 4).

Three-dimensional hydrogel cultures of patient-derived GB cells showed good viability and proliferation rates equivalent or superior to when they had been cultured as standard neurospheres. The hydrogel system also allowed the incorporation of the ECM mimetic peptides to reduce the effects of specific cell–ECM interactions [140,143,170,171]. In addition, the system described by other authors [148] provides a useful PEG-HA and PCL+/−BC/GEL-HA 3D in vitro mechanomimetic with a stiffness tunability, a model for elucidating the underlying mechanisms of GB progression in a more physiologically appropriate and controlled manner and assessing the efficacy of potential drug candidates [146,170,172]. In another similar study, the HA brain-mimicking hydrogel network resulted in significant dose-dependent changes in markers of glioma malignancies compared to unmodified 3D gelatin or PEG hydrogels [146,172,173]. The HA-modified hydrogel system provided a clear and reproducible extracellular microenvironment for studying the development of gliomas [173].

The scaffold Gliadel^®^ underwent clinical trials and has been FDA approved. It is a polyanhydride copolymer wafer loaded with carmustine or BCNU. This post-resection treatment is the only GB implantable device on the market and shows an effective bio performance with a few side effects [35,174]. Alternative systems have been developed with biopolymers such as silk fibroin, hyaluronic acid and heparin [67,107], such as formulating sponges loaded with SDF-1α chemoattractant cytokine that acts as tumor trap for CXCR4 receptor-positive cells. This strategy has also been explored in a recent study [175], using chitosan-based electrospun fibers charged with SDF-1α loaded PLGA nanoparticles for GB treatment. It was shown that a 7-day follow-up study of Fischer rats with implanted devices had no side effects in vivo [175]. Moreover, the nanofiber structure of the scaffold provided excellent fixation sites to aid the adhesion of human GB cells. Some improvement must be achieved to better shape the resection cavity and optimize the drug quantity that can reach sites of interest, in order to increase the bioperformance and efficacy of the system [175].

### 5.3. Systems for CNS Application

#### 5.3.1. Discussion of Hydrogels

HA-PDL hydrogels have been explored to repair brain tissue defects and showed a good bridging property, helping tissue ingrowth and vascularization in vitro and in vivo in Sprague–Dawley rat models [176]. In other studies, amino acid-based hydrogels have been prepared and improved tissue restructuration through angiogenesis and axonal growth in Sprague–Dawley rats models [177,178]. These formulated HA hydrogels also prevented glial scar formation by lowering glial cell proliferation also in Sprague–Dawley rats models [132]. Tam et al. [179] developed an HA-MC hydrogel to deliver NSPCs (neural stem/progenitor cells) that allow for OLG (oligodendrocyte) differentiation. In addition, respectively formulated HA nanocomposite hydrogels loaded with BDNF [180] and VEGF [181] in vitro SP embryo cell lines show a stable release of biological factors, allowing for cell survival and growth, and an HA-laminin hydrogel charged with SDF-1α in an in vitro and in vivo C57BL/6 mouse model enhanced the retention and migration of NPSC grafts post SDF-1α treatment in a signal-dependent manner through the SDF-1α-CXCR4 axis.

HA-poly(N-isopropylacrylamide) has shown to support cell survival and differentiation, have good biomimetic rheological properties, and stimulate the ECM network production [182]. Iron oxide (Fe_3_O_4_)-HA nanogels, intended for the treatment of Alzheimer’s disease via a theranostic tool containing a metal complex associated to HA nanogels, have shown a good biocompatibility to astrocyte cells and as a contrast agent of quality in MRI imaging in vitro [159]. This theranostic system could be a promising choice for neurodegenerative disease theranostics in vitro and in vivo [159].

#### 5.3.2. Discussion of Scaffolds

In other studies, developed scaffolds composed of biopolymers like collagen, alginate and PCL have shown an interesting bioperformance and biocompatibility. These scaffolds had mechanical properties, mostly with a Young modulus ranging from 0.1 to 10 kPa, which is in concordance with the brain tissue Young modulus interval [142,183,184] (Figure 4).

Moreover, it has been shown through in vitro and in vivo assays that NSCs embedded in HA collagen biomaterials can ameliorate the recovery of damaged facial nerves and the artificial conduction of NSCs may bring potential for the treatment of peripheral nerve damage. Aligned nanofibers allow for guiding the growth of neurites [142]. 

Diverse developed collagen-HA-based scaffolds were tested in vitro. It has been shown that their system promoted the differentiation of neural stem cells (NSCs) into neurons in vitro [183].

### 5.4. Systems for PNS Application

HA has been very successful in neural tissue engineering and supports the growth, differentiation and proliferation of neurites on a variety of substrates [151,185] (Figure 4). HA hydrogel amends the viability and proliferation of neural progenitor cells [151]. This indicates the potential therapeutic approaches for peripheral nerve regeneration and CNS therapies. HA hydrogels’ mechanical properties (Figure 4) have been adapted for the differentiation of neural progenitors, an up-and-coming strategy for neurodegenerative diseases treatment.

HA can be blended with different biopolymers, especially collagen, since they both enter in the composition of the ECM and are biocompatible and biomimetic when formulated in scaffolds. For example, some authors have used neural stem cells embedded in the HA/collagen conduit to regenerate a 5 mm facial nerve gap in a rabbit model [142]. In addition, a blend of HA with biodegradable synthetic polymers such as PLGA and poly-L-lysine has shown a great potential for the controlled delivery of drugs, targeting axonal regeneration after a spinal cord injury in vitro and in vivo [186].

The high biocompatibility of HA is crucial in reducing the inflammatory response produced by conductive polymers in nervous tissue engineering. For instance, PEDOT-doped HA nanoparticles are integrated into chitosan/gelatin scaffolds and exhibit excellent PC12 cell adhesion and growth, while pyrrole/HA conjugates mask conductive electrodes from adverse reactions of glial cells during implantation [187]. Nanofiber-aligned PCL/Gel/HA scaffolds have been shown to promote axon growth and elongation and help support intracellular communication [145]. Based on these results, the PCL/gel/HA composite scaffold is an excellent candidate for a biomimetic matrix for GB and tumor testing.

#### 5.4.1. Discussion of Conduits Systems

Many studies have developed composite HA-based conduits for nerve regeneration. Most of them performed experiments on preclinical models, such as SD rats, CD-1 mice and NZ rabbits, that helped validate these systems [142,147,185,188]. Additionally, some of the systems have not shown a significant regeneration. HA/collagen conduits have been developed but showed limited results with unmyelinated nerve fibers remaining; according to the authors, the scaffold needed to be amended since myelin degeneration and swelling can be observed [142]. For other authors, a similar system has shown a good stability, cell growth and adhesion and neurosphere development on the conduit scaffold, making possible the differentiation and nerve tissue regeneration [185]. 

#### 5.4.2. Discussion of Hydrogels

Some hydrogels have shown good results in terms of their biocompatibility, architectural properties, mechanical features and their bioperformance in vitro and in vivo. The sustained release of NGF from these chitosan and HA-based hydrogels was well-controlled [141,151,189]. The porosity and viscoelastic properties of their systems are interesting for cell attachment. Moreover, these hydrogels enhance neural regeneration and tissue repair but also cell differentiation and migration [141,151,189].

PDLLA/βTCP nerve conduits containing CS-HA/NGF hydrogels have also been described in the literature [147]. These devices enhanced nerve regeneration and myelination in contrast to the void PDLLA/βTCP nerve channels and autologous transplant group [147]. This suggests that injectable CS-HA/NGF hydrogels can successfully enhance nerve regeneration, and are therefore a good candidate in the field of neural tissue engineering [147].

## 6. Conclusions

HA is a promising material for GB, CNS and PNS injuries treatment due to its biomimetic, biomechanical, biocompatible and biodegradable properties, and the fact that it is tunable with chemical modifications and associable to other polymers makes it possible to create a simple scaffold for cell encapsulation, allowing for glial, neural cells and nerve fibers regeneration. It is also a promising material for more complex systems such as thermosensitive, (nano)composite systems for targeted drug delivery and local administration (Figure 5).

Indeed, treatments aimed to treat pathologies other than the CNS and PNS ones are available on the market using other administration routes, such as Orthovisc^®^, which is a topical preparation of highly modified HA that has shown a successful osteoarthritis (OA) treatment [190,191,192]. Hyalone^®^ treats osteoarthritis and targets lower back pain [192], Cartistem^®^ is used for ligament and cartilage degeneration including degenerative OA [193] and Hyalofast^®^ is utilized for chondral and osteochondral lesion treatment [194]. Hence, these systems can be adapted for their biomimetic properties and for better targeting of GB, CNS and PNS impairments.

In conclusion, HA-based devices present qualities illustrated in the Figure 5, like high biocompatibility, low invasiveness, controlled drug release, the reduction of tissue damage and permit, in most cases, the local administration of APIs. The efficacy of such systems is conditioned on the drug quantity initially put in the scaffold, and the size of the scaffold.

## Data Availability

Not applicable.

## Supplementary Materials

The following supporting information can be downloaded at: https://www.mdpi.com/article/10.3390/ijms232012174/s1. References [195,196,197,198,199,200] are cited in the supplementary materials.

## Abbreviations

BBB	Blood brain barrier
BC	Bacterial cellulose
BCNU	Carmustin or β-chloro-nitrosourea
BDNF	Brain-derived neurotrophic factor
CB	Cucurbit[n]uril
CED	Convection enhanced diffusion
CNS	Central nervous system
CS	Chitosan
CXCR4	C-X-C motif chemokine receptor 4
DOX	Doxorubicin
ECM	Extra cellular matrix
ELP	Elastin-like polypeptide
FDA	Food and Drug Administration
FU	focused ultrasound
GAG	Glycosaminoglycan
GB	Glioblastoma
GEL	Gelatin
GRAS	Generally recognized as safe
HA	Hyaluronic acid
LCGT	lower critical gelation temperature
MC	Methylcellulose
MRI	Magnetic resonance imaging
NGCs	Nerve guide channels
NGF	Nerve growth factor
NSCs	Neural stem cells
OLG	Oligodendrocyte
P407	Poloxamer 407
PCL	Polycaprolactone
PDLLA	Poly-d,l-lactic acid
PEG	Polyethylene glycol
PEOx	polyethylene oxide
PG	Propylene glycol
PNNs	Perineuronal nets
PNS	Peripheral nervous system
PPO	Poly propylene oxide
PXL	Paclitaxel
ROS	Reactive oxygen species
SDF-1α	Stromal cell-derived factor 1
TMZ	Temozolomide
UCGT	Upper critical gelation temperature
VEGF	Vascular endothelial growth factor
