# Supplementary material for: Hyaluronic Acid Scaffolds for Loco-Regional Therapy in Nervous System Related Disorders
Source: Int J Mol Sci. 2022 Oct 12;23(20):12174. doi: 10.3390/ijms232012174 (PMC9602826; doi:10.3390/ijms232012174)
Supplement: Supplementary file 1 [file ijms-23-12174-s001.zip › ijms-1903092-supplementary.pdf]

Table S1. Devices development for Glioblastoma application

Device	Matrix composition	Proposed mechanism	Study model (In vitro, in vivo, ex vivo)	Major Findings	Reference
**Hydrogel system**	HA-functionalized Methacrylate gelatin/poly(ethyl ene glycol) (PEG)4A	Mimic the microenvironment matrix for GBM cells	2-3D cultures, patient-derived GBM cells	HA-dose-dependent alterations in GBM malignancy-associated markers. HA can lead to a specific interaction in EGFR+ cells via CD44.	[Pedron et al., 2013] 10.1016/j.biomaterials.2013.06.024
**Composite hydrogel**	Collagen-Hyaluronan	Mimic the microenvironment matrix for GBM cells	3D culture, patient-derived GBM cells	Cell morphology, spreading and migration were influenced by collagen type and HA concentration.	[Rao et al., 2013] 10.1021/am402097j
**Hydrogels**	HA-Gelatin thiol-reactive PEG diacrylate (PEGDA)/PEG divinyl sulfone (PEGDVS) crosslinked	Mimic the microenvironment matrix for GBM cells and develop a better analytical analysis *in vitro*	Single-cells, 3D culture, U118, U87R cell lines	Potential to identify differences in the invasive behaviour of the cell and analyse the cellular response to biophysical signals in the extracellular environment.	[Heffernan et al., 2014] 10.1007/s10439-014-1223-1
**Composite scaffold**	Chitosan-HA scaffold	Mimic the microenvironment matrix for GBM cells	Single-cells, 2D culture, GSC GBM6 cell lines	Better cellular morphology, growth patterns, and malignant behaviour than adherent monolayers.	[Wang et al., 2016] 10.1002/adhm.201600684
**Micelles**	HA-Modified Micelles lauroyl-gemcitabine (Gem-C12) and honokiol (HNK) encapsulated	Nanocarriers for targeting drug delivery of combined chemotherapeutics	Single-cells, 3D culture, U87 cell line<br><br>Subcutaneous glioma-bearing mice; orthotopic xenograft GBM	Deep penetration into spheroids, endocytosis mediated by the CD44 receptor, and increased cytotoxicity of the combined treatment. Enhanced animal survival rate.	[Liu et al., 2018] 10.1021/acs.molpharmaceut.7b01035

**Thermosensitive hydrogel**	Doxorubicin/paclitaxel embedded Pluronic F127/HA hydrogel system (freeze-dried micelles)	Intratumoral administration of combined chemotherapy	Degradation rate, drug release<br><br>Injection force determination, Chicken meat model	Controlled release of both chemotherapeutics and great stability. The formulation also showed easy injectability.	[Rezazadeh et al., 2018] 10.4103/1735-5362.228918
**Hydrogel**	HA/ poly(ethylene glycol) (PEG)	Mimic the microenvironment matrix for GBM cells	3D culture, patient-derived GBM cells	Cell viability and proliferation rates are as good as or better than standard glioma spheres culture. Compatible results for use in molecular and cellular analyses.	[Xiao et al., 2018] 10.3791/58176
**Aerogel sponge**	Stromal cell derived factor-1α loaded in Silk fibroin-HA-Heparin	Chemoattractant loaded scaffold trap for brain tumor cells	Single-cells, NIH3T3 cell line	Cytocompatible material with adequate mean pore diameter (60 μm) and connectivity for welcoming cells. Texture similar to brain tissue (6–13 kPa Young's Modulus), slower biodegradation and 93% retention of SDF-1α.	[Najberg et al., 2019] 10.1016/j.carbpol.2020.116107
**Nanofibers scaffold**	Polycaprolactone (PCL) /gelatin (Gel) / hyaluronic acid (HA)	Mimic GBM extracellular matrix	Single-cells, U251 cell line	The topography and surface chemistry (hydrophilic) played an important role in the biomechanical, proliferative, and morphological properties of the cells.	[Unal et al., 2020] 10.3390/ma13112661
**Drug loaded Hydrogel**	Crosslinked-HA-adipic acid dihydrazide (ADH) loaded with human urotensin (hUII) peptide and doxorubicin (DOX) or temozolomide (TMZ)	Chemoattractant matrix to trap and eradicate GBM cells	Single-cells, U87MG cell line	Cell migration and invasion into the hydrogel in response to the hUII. Significant cytotoxicity of DOX-loaded hydrogels for U87MG cells.	[Kasapidou et al., 2021] 10.1039/d1sm01003d
**Drug-loaded hydrogel**	Peptide functionalized HA (HACF) cross-linked by	Drug delivery reservoir for resection cavity	Single-cells, patient-derived GBM cells	Biocompatible, adaptable, and adjustable to local tissue stiffness. 45% improvement in patient survival.	[Parkins et al., 2021] 10.1016/j.biomaterials.2021.120919

	cucurbit[8]uril (CB[8])		Patient-derived xenograft (PDX) rats		
**Hydrogel**	Methacryloyl-modified HA (HAMA)	Tissue engineering application	-	Suitable structure, porosity, and stiffness for tissue engineering purposes. Also, it showed to be resistant to collagenase biodegradation.	[Velasco-Rodriguez et al., 2021] 10.3390/ijms22136758
**Composite scaffold**	HA/gelatin (Gel) modified bacterial cellulose (BC)	Mimic GBM extracellular matrix	3D culture, U251 cell line	Good cell vitality, forming multi-layers and cell clusters in the scaffolds.	[Unal et al., 2021] 10.1007/s10570-020-03528-5
**Drug loaded Nanogel**	Lactoferrin (Lf)/phenylboronic acid (PBA)-functionalized HA nanogels crosslinked with disulfide-bond and doxorubicin hydrochloride (DOX)-loaded	Drug delivery and dual-targeting GBM	Single-cells and coculture, G422, bEnd.3 cell lines<br><br>SD rats - pharmacokinetics, ICR mice - biodistribution	Controlled release of the drug, effective BBB penetration, and precise glioma dual-targeting (PBA and HA), presenting strong cytotoxicity against G422 cells.	[Zhang et al., 2021] 10.1016/j.cjche.2021.08.029
**Polymer-drug conjugate**	HA-Hydrazone-DOX	GBM local treatment	2-3D culture GL261, U87MG, B16F10 cell lines GL261-bearing mice	Increased tumor cell mortality and inhibition of the tumor growth. Bio-responsive linker was crucial for the observed bioperformance.	[Malfanti et al., 2022] 10.3390/pharmaceutics14010124

Table S2. Systems developed for CNS injuries application

Device	Matrix composition	Proposed mechanism	Porosity connectivity diameter	Mechanical properties	Study model (In vitro, in vivo, ex vivo)	Major Findings	Reference
**Hydrogel**	HA –PDL hydrogel	Scaffold material for the repair of defects brain tissue	Good connectivity and adherence	-	• Human DRG cell line and NSCs<br>• 3 months old Sprague Dawley rats	After implantation, the polymer hydrogel rightly bridged the tissue defect, establishing a permissive interface with the host tissue to aid cell ingrowth and angiogenesis.	[Tian et al.2005] 10.1089/ten.2005.11.513
**Hydrogel**	HA-arginine-glycine-aspartic acid (RGD)	Tissue engineering for brain regeneration	-	-	• Sprague-Dawley rats, cortex implantation	HA-RGD hydrogel provided a structural, 3D continuity across the cortex and favoured reorganisation of local wound-repair cells, angiogenesis, and axonal growth.	[Cui et al., 2006] 10.1007/s10856-006-0615-7
**Hydrogel**	HA- Poly-D-lysine (PLL)/ nogo-66 receptor antibody (antiNgR)	Tissue engineering for nerve regeneration	10 to 100 μm<br><br>interconnected network	-	• Sprague-Dawley rats, lateral hemisection of spinal cord	Inhibited the glial scar formation, support angiogenesis, and promote axonal extension.	[Wei et al., 2007] 10.1002/jbm.b.31689

**Sponge-like scaffold**	Collagen type I and II/HA	Neurogenic induction of cells seeded into a construct for brain regeneration	80-200μm pore size(75% to 91%)	1-6.3kPa Young modulus	• 2-3D, primary cell culture	The scaffolds favour the differentiation of neural stem cells (NSCs) to neuronal cells in vitro.	[Wang et al., 2009] 10.1016/j.actbio.2009.03.033
**Gel**	HA	Matrix to reduce the marginal glial scarring	120 to 182 μm porosity	Tensile strength: 4.1-6.3 N/mm2	• Sprague-Dawley rats	Inhibited the glial scar formation by decreasing the thickness of gliosis and by reducing the number of the glial cells.	[Lin et al., 2009] 10.1016/j.wneu.2009.09.004
**Hydrogel-microspheres composite**	HA hydrogel / recombinant human brain-derived neurotrophic factor (BDNF) and vascular endothelial growth factor (VEGF)-poly(lactic-co-glycolic acid) microspheres loaded	Delivery system for NSCs	-	-	• 2-3D, primary and single-cells cultures (Sprague-Dawley embryos forebrain cells)	Stable releasing of the biofactors, promoting cells survival and growth. The soft property was suitable for CNS tissue. Biocompatible material, with great adhesion and cell proliferation.	[Wang et al., 2011] 10.1007/s11095-011-0452-3
**Hydrogel**	HA-Modified methylcellulose	NSPCs cells delivery vehicle	-	-	• Rat NSPCs cells	Increased oligodendrocytes differentiation.	[Tam et al.,2012] 10.1039/c2jm33680d

**Electrospun nanofiber scaffold**	HA-laminin-coated polycaprolactone (PCL) nanofibers	Neural tissue engineering for restore damaged tissue	Fiber diameters: 600–1200 nm	-	• 2-3D cell culture, eGFP neuronal SH-SY5Y cell line	The aligned nanofibers permitted the guidance of neurite outgrowth.	[McMurtrey, 2014] 10.1088/1741-2560/11/6/066009
**Hydrogel**	HA-laminin/SDF-1α loaded	Matrix to enhance neural transplant retention and migration delivery system	-	-	• 3D, primary cell culture; NPSCs<br>• C57BL/6 mice, brain injection	SDF-1α signalling critically mediates NPSC transplant chemotactic migration, and the matrix increases NPSCs transplant retention.	[Addington et al., 2017] 10.1016/j.matbio.2016.09.007
**Microporous annealed particle hydrogel**	HA/K-peptide/Q-peptide/RGD	Matrix to promote brain tissue repair after stroke	-	-	• C57BL/6, stroke-induced	Reduced inflammatory response following stroke and increased peri-infarct vascularization.	[Nih et al., 2017] 10.1002/adma.201606471
**Drug delivery scaffold**	SA and HA loaded device	Microenvironnement to embed stem cells in traumatic brain injury	200 µm to tens of µm	0.1 kPa to 1 kPa Young Modulus	• HUC-MSCs<br>• SD rats	NSCs embedded in HA collagen biomaterials may promote reinnervation of damaged facial nerves, and artificial conduction of NSCs may provide	[Zhang et al., 2008] 10.1039/C7TB03213G

						a potential treatment for peripheral nerve injury.	
**Hydrogel**	Glycidyl methacrylate-HA and Methacrylic anhydride-HA/Collagen I and laminin functionalized	Engineered scaffold for tissue regeneration	1 µm pore size	2-6kPa Young modulus	• Schwann Cells and dorsal root ganglia (DRGs) cell lines<br>• Rat isolated tissue (sciatic nerve, brain and spinal cord)	The material can be tuned to numerous soft tissues, and supported 3D axonal elongation of DRGs cultures.	[Spearman et al., 2020] 10.1002/jbm.a.36814.
**Hydrogel**	HA-galactose oxidase (GalOx)-horseradish peroxidase (HRP)/ bone mesenchymal stem cells (BMSC) and nerve growth factors (NGF) encapsulated	Implant or cell/drug delivery vehicle for tissue repair	<100um pore size<br>loose and porous structure	Low elastic modulus <0.1kPa	• 3D and single-cells models; BMSC cells<br>• Traumatic Brain Injury (TBI) model, C57BL/6 mice	Good biocompatibility, stability, and biodegradability. Suitable for cell survival and proliferation, accelerated brain repair process.	[Wang et al., 2022] 10.1016/j.mtbio.2021.100201
**Hydrogel**	HA-poly(N-isopropylacrylamide)	Hybrid interpenetrating polymer network as cell carrier for	-	0.1kPa-8kPa Elastic modulus	• Primary culture, NP cells<br>• Cartilage and intervertebral	The matrix supported the cell viability and the phenotype expression of the encapsulated cells.	[Guo et al., 2022] 10.1016/j.carbpol.2021.118828

		nucleus pulposus (NP) repair			disc (IVD) organ culture		
**Nanogel**	Iron oxide ($Fe3O4$)-HA	Theranostic material for Alzheimer's disease	–	-	• Single-cells culture, C8-D1A cell line	Non-cytotoxic matrix. Efficient generation of negative contrast in MRI.	[Chen et al., 2022] 10.1016/j.arabjc.2022.103748

Table S3. Systems developed for PNS injuries application

Device	Matrix composition	Proposed mechanism	Porosity and connectivity	Mechanical properties	Study model (In vitro, in vivo, ex vivo)	Major Findings	Reference
**Composite Conduit**	HA / collagen	Tissue engineering for nerve regeneration	Diameter 1.2 mm<br>Porosity 50 μm	-	• Rat SCs and neurospheres<br>• NSCs from neural cortex of SD rat embryos	Cultured rat Schwann cells and neurospheres grow *in vitro* on new artificial HA-based nerve conduits. After 3 weeks of culture, conduits stayed circular with a round lumen, and cell-conduits conserved their original structure.	[Sakai et al., 2007] PMID: 17502696
**Composite conduit**	HA / collagen	Tissue engineering for nerve regeneration	7 mm in length	-	• Normal adult New Zealand rabbits	NSC-embedded NT-3 HA-collagen composite scaffold, has shown that a number of nerve fibers were still unmyelinated. Degeneration and swelling of myelin lamellae were also visible.	[Zhang et al., 2008] 10.1186/1479-5876-6-67

**Composite Conduit**	HA-Silk Fibroin composite device	Tissue engineering for nerve regeneration	79% of porosity	G'/G': 6 to 7 viscoelasticity	• Rat Schwann cells (rSCs)<br>• Male CD-1 mice	Both HA and HA-SF scaffolds were received by the host with no residual immune response at 8 weeks. The results show that SF incorporation improves the mechanical properties of the material and leads to promising biocompatible conduits for tubing strategies.	[Gisbert et al., 2020]<br><br>10.1016/j.ijbiomac.2020.01.149
**Composite Conduit**	PDLLA/β-TCP/HA/CHS/NGF	Polymer based scaffold for controlled release of NGF	Semi-permeable, pore size less than 10 μm	13-60 kPa Elastic modulus	• PDLLA/β-TCP nerve conduits<br>• Sciatic nerve defect in SD rat	Substantial enhancements in nerve regeneration were noticed after using the PDLLA/β-TCP/HA/CHS/NGF NGCs based on the different assessments. In vivo studies show that the PDLLA/ β-TCP/ HA/ CHS/NGF sustained-release NGCs can significantly stimulate peripheral nerve regeneration, and the effect is comparable to that of autograft.	[Yan et al., 2021]<br><br>10.1007/s11595-021-2450-6
**Scaffold**	PEDOT-doped HA NPs/ CS / Gel matrix	Tissue engineering for nerve regeneration	200– 300 μm pore size<br><br>83-92% porosity	13-60 kPa Elastic modulus	• PC12 cell line	8% PEDOT-HA/Cs/Gel scaffold had a higher cell adhesive efficiency and cell viability in comparison to the other conductive scaffolds. It also has shown a higher expression of synapse growth genes of GAP43 and SYP compared	[Wang et al., 2017]<br>10.1016/j.msec.2016.10.029

						with the Cs/Gel control group.	
**Hydrogel**	HA-CS composite system	Hydrogels for nerve growth factor(NGF) sustained release and nerve regeneration	Porosity: 20 to 100μm	2 to 4 kPa Elastic modulus	• RSC96 rat SCs and PC12 cell lines<br><br>• Healthy adult Sprague-Dawley (SD) rats	• The good mechanical, porous, and swelling properties play a synergistic role in enhancing nerve regeneration. The CS-HA/NGF hydrogels through a sustained release are favorable for promoting cell adhesion, spreading, and differentiation<br><br>• Chitosan and HA inhibited extraneural scarring, promoted nerve regeneration, raised the nerve conduction velocity and amended the recovery of nerve function.	[Xu et al., 2016] 10.1177/0883911516662068<br><br>[Li et al., 2018] 10.3892/mmr.2018.8388

						Combined together they enhance neural regeneration and repair.	
**Hydrogel**	HA-CS/NGF hydrogel	Injectable hydrogels aimed to sustained release of nerve growth factor (NGF)	Pore size 53-73μm<br><br>Porosity(82-87%)	2.3-4.5kPa Elastic modulus	• BMMSCs, RSC96 rat SCs cells and PC12 cell line	PDLLA/β-TCP nerve conduits formulated with CS-HA/NGF hydrogels improved the axon regeneration and myelination, contrasted to the PDLLA/β-TCP hollow nerve conduits and the autograft group. These findings imply that the CS-HA/NGF injectable hydrogel can successfully upgrade nerve regeneration, hence, it is a good candidate in the field of neural tissue engineering.	[Xu et al., 2022] 10.1016/j.compositesb.2021.109509
